# Progression of Motor and Cognitive Functions in Isolated REM Sleep Behavior Disorder: A 7‐Year Prospective Matched Cohort Study

**DOI:** 10.1002/mds.70346

**Published:** 2026-05-01

**Authors:** Li Zhou, Yaping Liu, Bei Huang, Jing Wang, Shi Tang, Yuhua Yang, Siyi Gong, Steven W.H. Chau, Joey W.Y. Chan, Mandy W.M. Yu, Jessie C.C. Tsang, Shirley Xin Li, Siu Ping Lam, Vincent C.T. Mok, Karen K.Y. Ma, Anne Y.Y. Chan, Jihui Zhang, Yun Kwok Wing

**Affiliations:** ^1^ Department of Psychiatry, Faculty of Medicine The Chinese University of Hong Kong Shatin, N.T., Hong Kong SAR China; ^2^ Li Chiu Kong Family Sleep Assessment Unit, Department of Psychiatry, Faculty of Medicine The Chinese University of Hong Kong Shatin, N.T., Hong Kong SAR China; ^3^ Department of Medicine Division of Pulmonary, Critical Care, and Sleep Medicine, Icahn School of Medicine at Mount Sinai New York USA; ^4^ Center for Sleep and Circadian Medicine, The Affiliated Brain Hospital Guangzhou Medical University Guangzhou China; ^5^ Li Ka Shing Institute of Health Sciences, Faculty of Medicine, The Chinese University of Hong Kong Shatin, Hong Kong SAR China; ^6^ Department of Psychology The University of Hong Kong Pokfulam, Hong Kong SAR China; ^7^ The State Key Laboratory of Brain and Cognitive Sciences The University of Hong Kong Pokfulam, Hong Kong SAR China; ^8^ Department of Medicine and Therapeutics, Faculty of Medicine The Chinese University of Hong Kong Shatin, Hong Kong SAR China; ^9^ Gerald Choa Neuroscience Institute The Chinese University of Hong Kong Hong Kong SAR China

**Keywords:** phenoconversion, progression, REM sleep behavior disorder, α‐synucleinopathies

## Abstract

**Background:**

Although clinical markers (eg, motor and cognitive impairment) in isolated rapid eye movement sleep behavior disorder (iRBD) are associated with faster phenoconversion, their longitudinal trajectory patterns (linear or nonlinear) remain unclear. Additionally, evidence regarding the magnitude of neurodegenerative risk in iRBD compared to non‐RBD individuals remains limited.

**Objectives:**

The aim was to investigate longitudinal changes in clinical markers and assess the magnitude of neurodegenerative risk in iRBD versus non‐RBD subjects.

**Methods:**

In this prospective matched cohort study, video‐polysomnography‐confirmed iRBD patients and age‐ and sex‐matched non‐RBD subjects were followed every 1.5–2 years to evaluate neurodegenerative outcomes and markers. Longitudinal marker changes and neurodegenerative risk between groups were compared.

**Results:**

One‐hundred thirty‐three iRBD patients and 101 non‐RBD subjects were followed for a mean of 6.9 ± 2.9 years. Parkinsonism‐first converters were associated with motor dysfunction, whereas dementia‐first converters were associated with cognitive, motor, olfactory, and color vision dysfunctions. Motor and global cognitive functions exhibited nonlinear progression in iRBD, with marked acceleration before parkinsonism or dementia diagnosis. Conversion rates in iRBD patients were 4.7% at 3 years, 30.1% at 7 years, and 79.5% at 13 years, exceeding those in non‐RBD subjects (3% at 3 years and 16.1% at 13 years). iRBD patients had a higher neurodegeneration risk (hazard ratio [95% confidence interval [CI]: 9.6 [3.8, 23.8], *P* < 0.001).

**Conclusions:**

Motor and global cognition demonstrate nonlinear accelerating progressions during iRBD phenoconversion. iRBD carries a nearly 10‐fold higher risk of future neurodegeneration than non‐RBD subjects. These findings highlighted a critical window for risk stratification and early intervention before neurodegenerative disease onset in iRBD. © 2026 The Author(s). *Movement Disorders* published by Wiley Periodicals LLC on behalf of International Parkinson and Movement Disorder Society.

Isolated rapid eye movement (REM) sleep behavior disorder (iRBD) is a parasomnia affecting 1%–1.5% middle‐aged and older adults, characterized by recurrent dream‐enactment behaviors (DEBs) and REM sleep without atonia (RSWA).^1^, ^2^, ^3^ Currently, iRBD is regarded as a specific prodromal stage of α‐synucleinopathies, namely Parkinson's disease (PD), dementia with Lewy bodies (DLB), and multiple system atrophy (MSA), along with a long disease trajectory until phenoconversion.^4^, ^5^ Previous studies have indicated that a series of neurodegenerative markers, including motor dysfunction, cognitive decline, olfactory loss, color vision impairment, daytime sleepiness, are present at this prodromal stage, whereas some of these clinical markers are associated with a faster phenoconversion in iRBD.^5^, ^6^, ^7^, ^8^, ^9^, ^10^, ^11^ Additionally, a previous study reported that different markers exhibited distinct temporal changes during iRBD disease course.^8^ Motor symptoms appeared to follow a nonlinear trajectory, with accelerated progression shortly before phenoconversion.^8^ However, it remains unclear whether other nonmotor markers also demonstrate similar nonlinear patterns during disease progression. The evidence for the nonlinear trajectories of markers before phenoconversion remains limited compared to non‐RBD subjects. A better understanding of the longitudinal changes in these markers may improve risk stratification and help define the optimal time window for future neuroprotective interventions.

Studies across diverse ethnic populations have shown that iRBD patients carry a high risk of phenoconversion.^5^, ^7^, ^12^, ^13^, ^14^, ^15^, ^16^, ^17^, ^18^, ^19^, ^20^ A meta‐analysis further reported that over 90% iRBD patients eventually develop a neurodegenerative disease within 14 years.^21^ Although the elevated neurodegenerative risk in iRBD patients is well established, the evidence regarding the magnitude of neurodegenerative risk in iRBD patients compared to non‐RBD subjects remains limited. Therefore, in this prospective cohort study, we aimed to (1) investigate the longitudinal patterns of change in a series of clinical markers, including both motor and nonmotor markers, in iRBD disease trajectory compared to matched non‐RBD subjects; (2) evaluate the magnitude of neurodegenerative risk among iRBD patients compared to matched non‐RBD subjects.

## Methods

### Study Design and Subjects

This was a prospective matched cohort study. iRBD patients and age‐ and sex‐matched non‐RBD subjects were recruited from our ongoing RBD cohort study with a regular follow‐up interval of 1.5–2 years in the Li Chiu Kong Family Sleep Assessment Unit, a major sleep center in Hong Kong. Non‐RBD subjects in our cohort were recruited from community and sleep clinic.^9^, ^22^ Details of inclusion criteria for subjects are provided in the Supporting Information Methods.

### Measurements

Self‐reported questionnaires were used to assess lifestyle, excessive daytime sleepiness (Epworth Sleepiness Scale), mood symptoms (Hospital Anxiety and Depression Scale, HADS), and RBD symptoms (RBD questionnaire‐Hong Kong, RBDQ‐HK) at baseline and each follow‐up visit.^23^, ^24^ Clinical comorbidities at baseline were collected from the Clinical Management System and supplemented by structured clinical interviews.^9^, ^23^ A battery of neurological assessments, including motor, cognition, olfactory, autonomic function, color vision, were administered during baseline and each follow‐up visit. The total likelihood ratio (LR) and probability of prodromal PD were calculated based on the updated Movement Disorder Society (MDS) research criteria (2019).^25^ Additional details on these measurements are provided in the Supporting Information Methods.

### Outcome Follow‐Up

All subjects were followed up at our sleep center to evaluate neurodegenerative outcomes, with detailed outcome confirmation described in our previous publications.^26^ Briefly, subjects who had the signs of clinical parkinsonism or cognitive impairment would be referred to neurologists, geriatricians, or psychogeriatricians for further evaluation of the confirmation of neurodegenerative disorders based on the clinical criteria, including UK PD Society Brain Bank Diagnostic Criteria for PD,^27^ DLB Consortium Criteria for DLB,^28^ or MSA.^29^ Generally, the “1‐year rule” is used to differentiate DLB from PD dementia (PDD): if dementia onset occurs within 1 year of parkinsonism, the diagnosis is DLB; if it occurs later, the diagnosis is PDD. In this study, PD was regarded as parkinsonism‐first converter, whereas DLB was regarded as dementia‐first converter.^26^ Patients with MSA diagnosis were included only in the neurodegenerative risk analysis. Besides, all neurodegenerative diagnosis or death could be retrieved from clinical medical system, which is an electronic database covering over 95% of the healthcare records in Hong Kong. Because other types of dementia were diagnosed in non‐RBD subjects, we used the term “neurodegenerative risk” rather than “α‐synucleinopathy risk” for comparison.

### Statistical Analysis

Detailed statistical analyses are provided in the Supporting Information Methods. Briefly, continuous and categorical variables were summarized as mean ± standard deviation or n (%). Kaplan–Meier survival curves and log‐rank tests were used to compare neurodegenerative risk between groups. Cox proportional hazards regression models were applied to estimate hazard ratio (HR) and 95% confidence interval (CI). Longitudinal changes in neurodegenerative markers between groups were analyzed using generalized estimating equation (GEE) models. To estimate the trajectory of neurodegenerative markers, generalized additive model was employed using the R package *mgsv*.^30^, ^31^, ^32^ The effective degree of freedom (EDF) was estimated by the models and represented the degree of nonlinearity. EDF greater than 1 but less than 2 indicates a weakly nonlinear relationship, whereas EDF greater than 2 indicates a highly nonlinear relationship.

For mortality cases, the date of death was used to calculate the follow‐up duration in the survival analysis, whereas the date of the follow‐up visit with neurodegenerative marker assessment was used for calculating the time duration in the longitudinal marker analysis. All statistical analysis was performed using *IBM SPSS* version 27.0 (Armonk, NY: IBM Corp) and *R project* (version 4.3.2). Missing data required for the statistical analyses were excluded. A two‐sided *P‐*value <0.05 indicated statistically significant. The Benjamini–Hochberg procedure was employed to adjust for multiple comparison, and a false‐positive rate of less than 5% indicated statistically significant.

## Results

### Demographics, Clinical Features, and Neurodegenerative Outcomes at Baseline

**FIG. 1:**
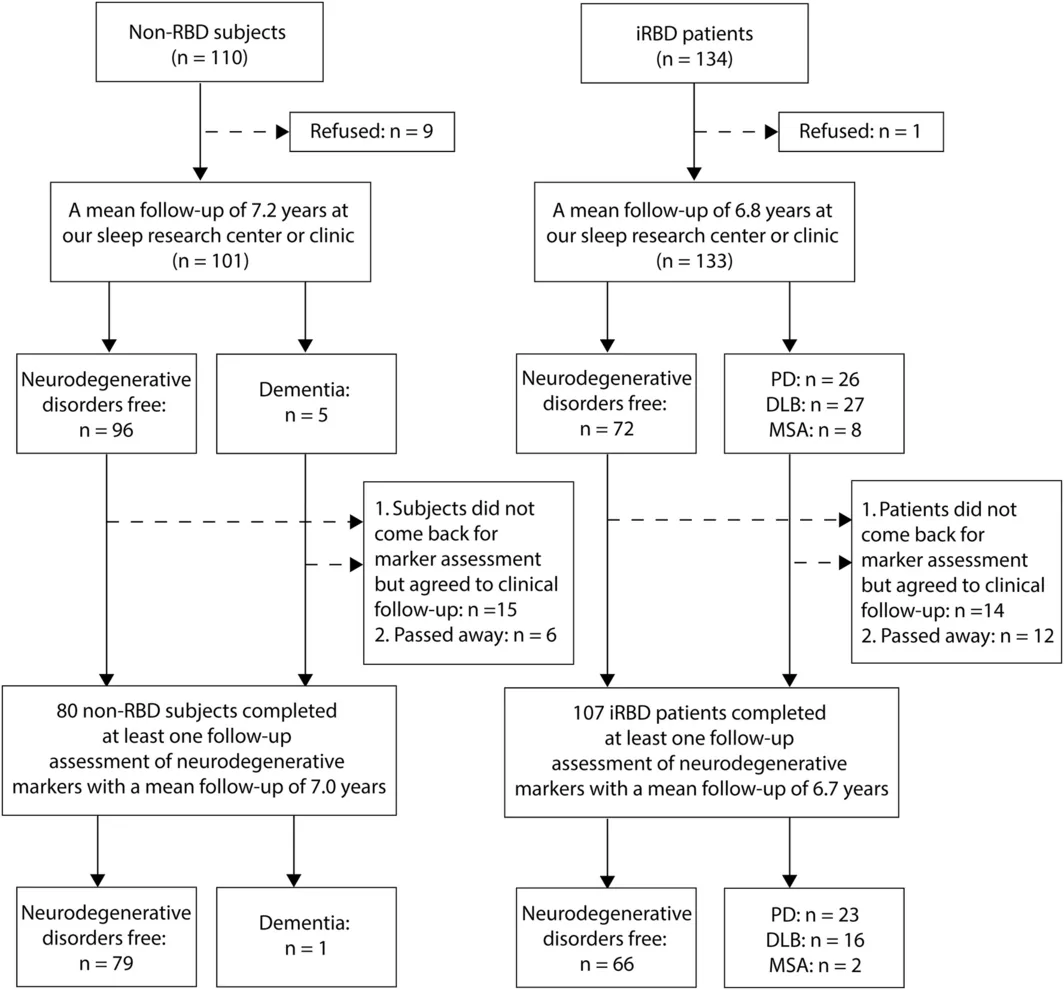
Flowchart of this study. DLB, dementia with Lewy bodies; iRBD, isolated rapid eye movement (REM) sleep behavior disorder; PD, Parkinson's disease; MSA, multiple system atrophy.

A total of 134 iRBD patients and 110 non‐RBD subjects were eligible for this prospective cohort study. Ten subjects (1 iRBD patient and 9 non‐RBD subjects) refused further follow‐up (Fig. 1). Finally, 133 iRBD patients and 101 non‐RBD subjects were followed up for neurodegenerative outcome evaluation. Regarding the assessment of neurodegenerative markers, 47 subjects (29 subjects only agreed to clinical follow‐up and 18 subjects passed away) did not complete the follow‐up assessment. Thus, 187 subjects completed at least two assessments on neurodegenerative markers, including 107 iRBD patients (mean age: 65.7 ± 6.9 years; 78.5% male) and 80 non‐RBD subjects (66.0 ± 7.4 years; 67.5% male). The mean number of follow‐up visits was 3.6 ± 1.5 visits (range: 2–8 visits). Figure S1 shows the percentage of data available for each neurodegenerative marker across 435 visits in iRBD patients and 229 visits in non‐RBD subjects. Subject characteristics between two groups at baseline are listed in Table 1. The age at baseline, sex, and education between iRBD patients (mean age: 66.3 ± 7.1 years, 75.9% male) and 101 non‐RBD subjects (66.7 ± 9.4 years, 70.3% male) were similar. The mean age at RBD onset in iRBD patients was 61.2 ± 7.3 years. The mean follow‐up duration in iRBD patients and non‐RBD subjects was 6.8 ± 3.1 years (range: 0.89–14.6 years) and 7.2 ± 2.9 years (0.91–14.4 years), respectively.

**TABLE 1:** Demographics, lifestyles, and clinical features at baseline

	Non‐RBD subjects (n = 101)	iRBD patients (n = 133)
Age at baseline, mean (SD)	66.7 (9.4)	66.3 (7.1)
Age at RBD onset, mean (SD)	‐	61.2 (7.3)
Male, n (%)	71 (70.3)	101 (75.9)
RBD duration at baseline since RBD onset, year, mean (SD)	‐	5.1 (3.4)
RBD duration at baseline since diagnosis, year, mean (SD)	‐	0.47 (1.6)
Follow‐up duration, year, mean (SD)	7.2 (2.9)	6.8 (3.1)
Education, secondary or above, n (%)	63 (62.4)	82 (61.7)
Lifestyle		
Smoking	n = 87	n = 110
Never	73 (83.9)	73 (66.4)
Former	10 (11.5)	17 (15.5)
Current	4 (4.6)	20 (18.2)
Alcohol drinking	n = 86	n = 110
Never	46 (53.5)	58 (52.7)
Former	4 (4.7)	14 (12.7)
Current	36 (41.9)	38 (34.5)
Coffee consumption	n = 87	n = 110
Never	39 (44.8)	51 (46.4)
Former	1 (1.1)	3 (2.7)
Current	47 (54.0)	56 (50.9)
Psychiatric disorders, lifetime	21 (20.8)	37 (27.8)
Antidepressant usage, lifetime	18 (17.8)	29 (21.8)
Constipation, lifetime	18 (17.8)	49 (36.8)

*Note*: Data are presented as mean (standard deviation) or n (%). The bolded *P‐*values indicated <0.05.

Abbreviations: RBD, rapid eye movement (REM) sleep behavior disorder; iRBD, isolated REM sleep behavior disorder; SD, standard deviation.

**FIG. 2:**
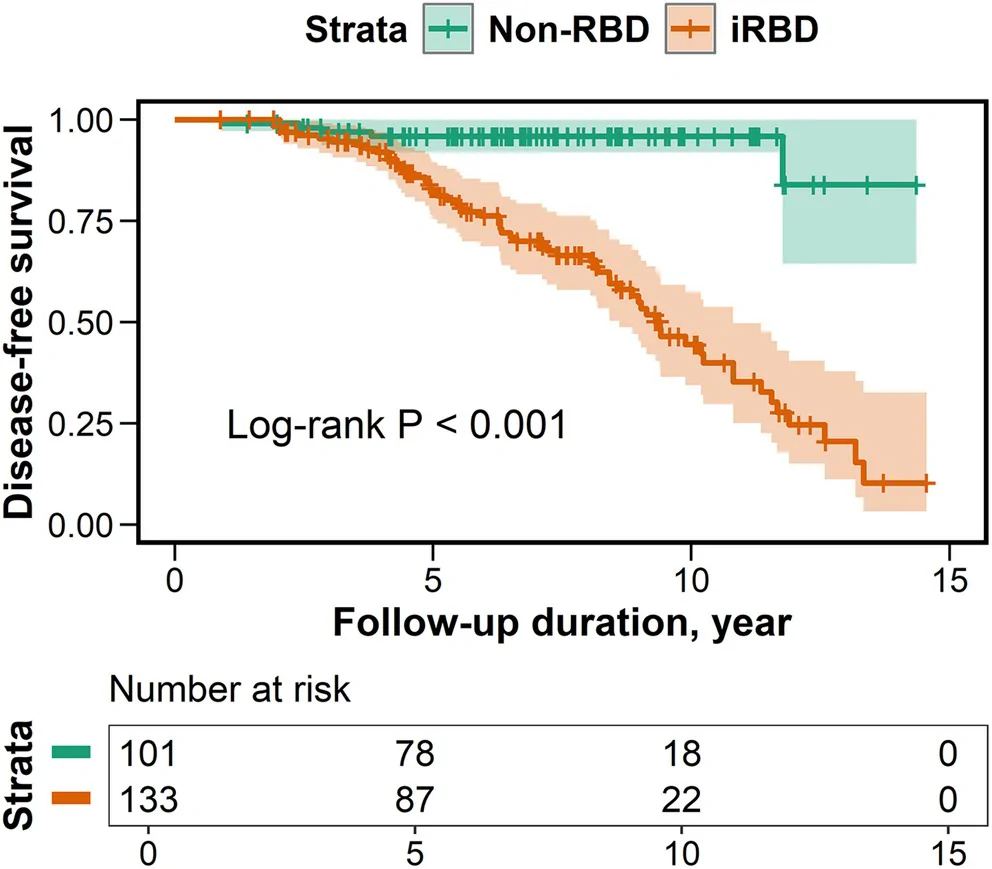
Kaplan–Meier curve for overall conversion rate in iRBD and non‐RBD subjects. iRBD, isolated rapid eye movement (REM) sleep behavior disorder. [Color figure can be viewed at wileyonlinelibrary.com]

Of 133 iRBD patients, 61 converted to α‐synucleinopathies, including 26 PD, 27 DLB, and 8 MSA, whereas 5 out of 101 non‐RBD subjects were diagnosed with dementia (2 vascular dementia, 1 mixed dementia, 2 dementia types not specified). The conversion rate of iRBD to neurodegeneration was 4.7% at 3 years, 17.9% at 5 years, 30.1% at 7 years, 55.6% at 10 years, and 79.5% at 13 years, which was higher than that of non‐RBD subjects (3% at 3 years and 16.1% at 13 years) (Fig. 2). iRBD patients showed a nearly 10‐time higher conversion risk of future neurodegeneration compared to non‐RBD subjects (HR [95% CI]: 9.6 [3.8, 23.8], *P* < 0.001).

### Longitudinal Change in Neurodegenerative Markers among Non‐RBD and iRBD

Compared to non‐RBD subjects, iRBD patients had significant declines in the *z* scores of RBDQ‐HK (time*group interaction: −0.05 [−0.08, −0.02], *P*_adjusted_ < 0.001), Olfactory Identification Test (−0.05 [−0.09, −0.02], *P*_adjusted_ < 0.001), and decline in HK‐MoCA (−0.05 [−0.09, −0.01], *P*_adjusted_ = 0.046), but significant increases in UPDRS‐Part III (0.07 [0.03, 0.11], *P*_adjusted_ < 0.001) and Grooved Pegboard Test (nondominant hand) (0.06 [0.02, 0.11], *P*_adjusted_ = 0.03) (Table S1). The changes of progression in other features were similar between the two groups. Due to the limited number of subjects completing the UMSARS‐Part III, only group difference was estimated in the model. Regarding MDS likelihood ratio of prodromal PD (excluding RBD item), iRBD patients had a high level of total LR and estimated probability of prodromal PD at baseline and a significant increase during follow‐up than non‐RBD subjects.

### Demographics and Clinical Features Among Nonconverters, Parkinsonism‐First, and Dementia‐First Converters at Baseline

Dementia‐first converters were of older age at baseline (70.2 ± 6.6 vs. 65.0 ± 6.9 vs. 65.0 ± 6.0, *P* = 0.02) and marginally older age at onset (64.2 ± 6.9 vs. 60.2 ± 7.5 vs. 59.5 ± 5.9, *P* = 0.084) than nonconverters and parkinsonism‐first converters, respectively (Table S2). In addition, parkinsonism‐first converters had a higher total LR of prodromal PD than nonconverters and dementia‐first converters (*P* = 0.035) at baseline. Interestingly, parkinsonism‐first converters and dementia‐first converters had similar levels of total LR and estimated probability of prodromal PD at phenoconversion.

To further understand the factors contributing to the baseline difference in LR of prodromal PD between parkinsonism‐first and dementia‐first converters (after removing polysomnography (PSG)‐confirmed RBD from the calculation), we performed additional exploratory analyses comparing baseline neurodegenerative markers between the two groups (Table S3). Our results showed that parkinsonism‐first converters had higher Epworth Sleepiness Scale score and marginally lower Olfactory Identification Test score, whereas dementia‐first converters demonstrated lower HK‐MoCA score. Other markers are comparable between the two groups.

### Longitudinal Change in Neurodegenerative Markers Among Nonconverters, Parkinsonism‐First, and Dementia‐First Converters

Parkinsonism‐first converters had a marginally faster increase in *z* scores of UPDRS‐Part III (time*group interaction: 0.14 [0.03, 0.25], *P*_adjusted_ = 0.08) than nonconverters (Table S4). Interestingly, parkinsonism‐first converters had higher total LR of prodromal PD at baseline (0.30 [0.02, 0.58], *P* = 0.03) but a similar progression rate during follow‐up when compared to the nonconverters.

Dementia‐first converters had a significant decline in *z* scores of HK‐MoCA (−0.14 [−0.23, −0.06], *P*_adjusted_ < 0.001) and Olfactory Identification Test (−0.08 [−0.13, −0.03], *P*_adjusted_ < 0.001), but increases in *z* scores for total errors on the hue test (0.15 [0.05, 0.26], *P*_adjusted_ < 0.001), UPDRS‐Part III (0.15 [0.04, 0.25], *P*_adjusted_ = 0.03), and Color Trail Test 2a (0.18 [0.03, 0.33], *P*_adjusted_ = 0.04) and 2b (0.16 [0.02, 0.30], *P*_adjusted_ = 0.04) than nonconverters (Table S5). Interestingly, dementia‐first converters had lower levels of total LR and estimated probability of prodromal PD at baseline but significant increase during the follow‐up than nonconverters.

### The Dynamic Changes in Neurodegenerative Markers During Disease Trajectory

**FIG. 3:**
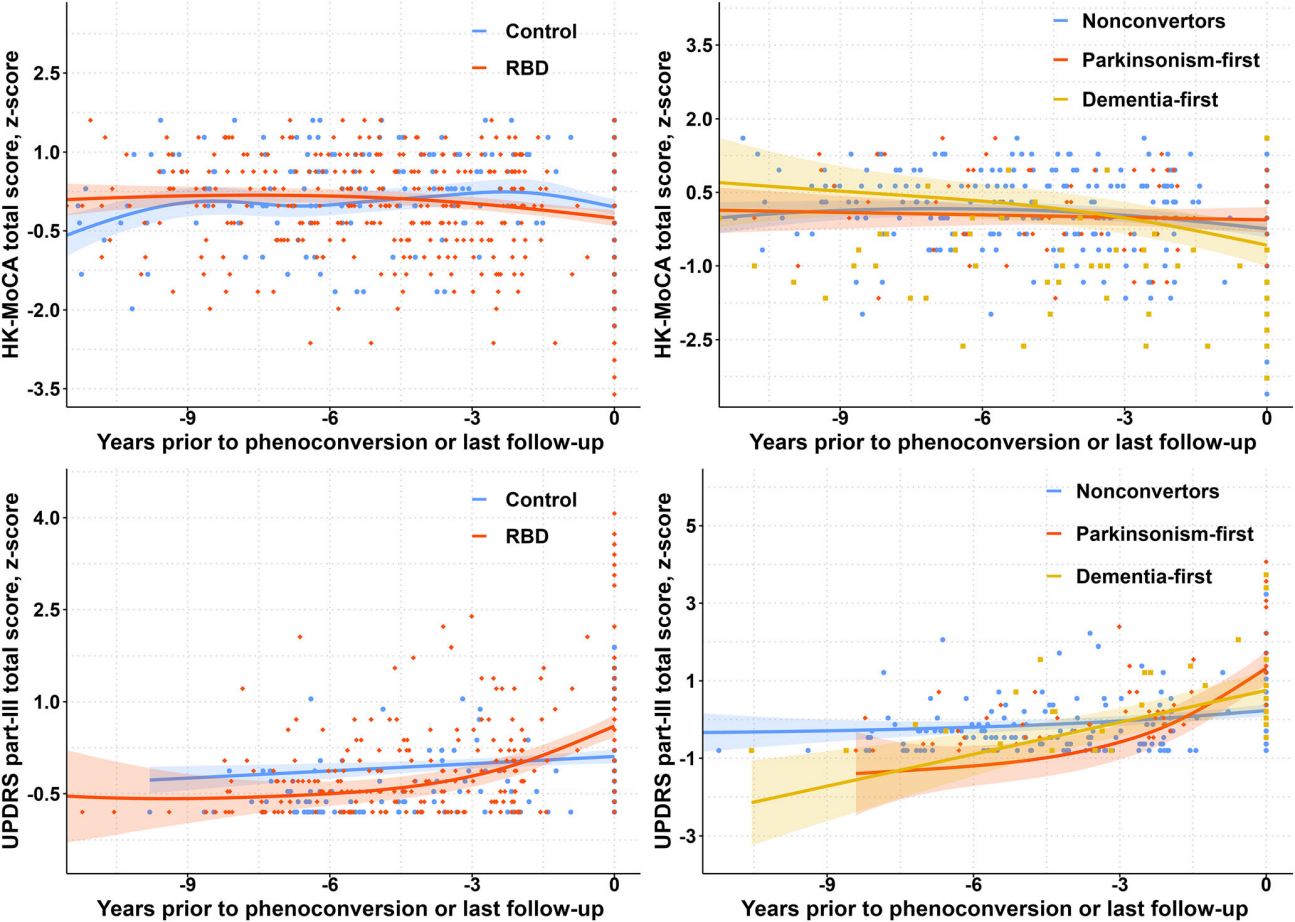
The dynamic changes in HK‐MoCA and UPDRS Part‐III among groups during disease progression. HK‐MoCA, Hong Kong Montreal Cognitive Assessment; UPDRS‐Part III, Unified Parkinson's Disease Rating Scale Part III. [Color figure can be viewed at wileyonlinelibrary.com]

The generalized additive model results showed that non‐RBD subjects had a nonlinear fluctuation in HK‐MoCA without a significant decline (EDF = 4.5, *P* = 0.02) (Table S6 and Fig. 3), whereas iRBD patients (EDF = 2.1, *P* < 0.001) and dementia‐first converters (EDF = 1.6, *P* = 0.009) had nonlinear declines in HK‐MoCA (Tables S6 and 2; Fig. 3). In addition, UPDRS‐Part III showed a nonlinear increase in iRBD patients (EDF = 2.7, *P* < 0.001) and parkinsonism‐first converters (EDF = 2.4, *P* < 0.001) but a linear increase in non‐RBD subjects (EDF = 1.0, *P* = 0.008). Regarding other markers, including Color Trail Test, Olfactory Identification Test, hue test, Grooved Pegboard Test (dominant and nondominant hands), and systematic and diastolic blood pressure drop, more linear changes over time across groups were shown (Tables S6 and 2; Figs. S2–S5).

**TABLE 2:** The correlation of neurodegenerative markers changes with time among nonconverters, parkinsonism‐first, and dementia‐first converters

	Nonconverters		Parkinsonism‐first converters		Dementia‐first converters	
	EDF	*P‐*value	EDF	*P‐*value	EDF	*P‐*value
HK‐MoCA, total, *z* score	2.3	**0.009**	1.0	0.54	1.6	**0.009**
UPDRS‐Part III, *z* score	1.8	**0.002**	2.4	**<0.001**	1.0	**<0.001**
Color trail 2a, sec, *z* score	2.2	**0.004**	1.6	0.62	1.0	0.13
Color trail 2b, sec, *z* score	1.2	0.05	1.0	0.05	1.0	**0.003**
ROCF, delay recall, *z* score	1.0	0.60	1.5	0.65	1.5	0.11
OIT, correct score, *z* score	1.7	0.06	1.0	0.20	1.4	0.12
FarnswortheMunsell 100 hue test, total error score, *z* score	1.0	**0.03**	1.0	**0.03**	1.0	0.37
Grooved Pegboard Test, dominant, sec, z‐score	1.5	**0.02**	1.0	0.08	1.0	**0.03**
Grooved Pegboard Test, nondominant, sec, z‐score	1.0	**<0.001**	1.6	0.06	1.5	0.05
UMSARS‐Part III						
Systolic blood pressure drop, mmHg, *z* score	1.2	**0.04**	1.0	0.21	1.0	0.14
Diastolic blood pressure drop, mmHg, *z* score	1.0	**0.009**	2.0	**0.03**	1.0	**0.012**

*Note*: The models were generated by generalized additive models adjusting for age at baseline and gender. Individual subjects were included as a random effect in the models. The bolded *P‐*values indicated <0.05.

Abbreviations: EDF, effective degrees of freedom; HK‐MoCA, Hong Kong Montreal Cognitive Assessment; UPDRS‐Part III, Unified Parkinson's Disease Rating Scale Part III; ROCF, Rey‐Osterrieth Complex Figure Test; OIT, Olfactory Identification Test; Sec: second; UMSARS‐Part III, Unified Multiple System Atrophy Rating Scale Part III.

Figure 3 displays the estimated dynamic changes in global cognition and motor symptom scores during the disease trajectory. For the global cognition, the HK‐MoCA score exhibited a relatively sharp decline before the neurodegenerative diagnosis among dementia‐first converters. In terms of the motor symptoms, parkinsonism‐first converters showed an increase in the UPDRS‐Part III score in the early years and a significant acceleration before neurodegenerative diagnosis.

### Comparison of Neurodegeneration Between Community and Clinical Non‐RBD Subjects

To address potential differences in neurodegeneration between clinical and community non‐RBD subjects in our study, we conducted additional analyses by comparing these two non‐RBD groups. Among clinical non‐RBD subjects, 94.3% had obstructive sleep apnea(of whom 78% received treatment), and 9.4% had insomnia. Table S7 shows that the two subgroups were similar in demographics, lifestyle factors, and prevalence rate of constipation. Nevertheless, clinic‐based non‐RBD subjects had a higher prevalence of lifetime psychiatric disorders, but their current anxiety and depression symptoms, as measured by the HADS, were comparable between community and clinical non‐RBD subjects. Regarding neurodegenerative risk, no significant differences were observed between the two control groups (Fig. S6). Additionally, longitudinal analyses of neurodegenerative markers revealed no differences between the groups (Table S8).

## Discussion

This prospective matched cohort study comprehensively investigated the nonlinear trajectories of motor and nonmotor clinical markers and, to our knowledge, for the first time evaluated the magnitude of neurodegenerative risk in iRBD patients compared to non‐RBD subjects. Our study demonstrated that the conversion rate of iRBD was 4.7% at 3 years, 17.9% at 5 years, 30.1% at 7 years, 55.6% at 10 years, and 79.5% at 13 years, which was higher compared to non‐RBD subjects (3% at 3 years and 16.1% at 13 years) in Hong Kong Chinese iRBD population. iRBD patients exhibited a nearly 10‐time higher conversion risk to future neurodegeneration compared to non‐RBD subjects. Additionally, parkinsonism‐first converters were associated with motor dysfunction, whereas dementia‐first converters were associated with cognitive, motor, olfactory, and color vision dysfunctions. Table S9 summarizes that the neurodegenerative markers most strongly associated with parkinsonism‐first and dementia‐first converters. There is a significant nonlinear progression of motor and global cognitive functions along the iRBD disease trajectory, with a markedly accelerated deterioration course before the diagnosis of parkinsonism or dementia. When compared to nonconverters, dementia‐first converters had a significant increase in total LR of prodromal PD during follow‐up, despite having a lower level at baseline. Conversely, parkinsonism‐first converters had a higher level of total LR of prodromal PD at baseline but exhibited a similar progression rate to that of nonconverters during the follow‐up.

Our study found that various neurodegenerative markers exhibited differential patterns of longitudinal changes during the disease course, particularly motor and global cognitive dysfunction with a nonlinear trajectory. A previous study also suggested that the motor dysfunction was progressive in a nonlinear fashion among iRBD patients.^8^ In addition, our study demonstrated a nonlinear progression of global cognition in iRBD patients and dementia‐first converters, which was similar to the findings in patients with PD or dementia diagnosis.^33^, ^34^, ^35^ As iRBD patients were already found to have positive pathological α‐synuclein in the cerebrospinal fluid, it might reflect the accumulation of α‐synuclein aggregates in the brain.^36^ The possible explanation for the nonlinear change might be attributed to the cumulative pathological burden leading to the breakdown of underlying neural circuitry mechanism at certain threshold.^33^

Our study showed that dementia‐first converters, with a later clinical presentation and marginally later onset of RBD,^37^ exhibited a significant increase in total LR of prodromal PD during follow‐up but a lower level at baseline than nonconverters. Conversely, parkinsonism‐first converters had a higher level of total LR of prodromal PD at baseline but did not exhibit a significant change during follow‐up. These differential trajectories for the parkinsonism‐ and dementia‐first conversion are intriguing. It might be that the current criteria for estimating the prodromal PD risk may not be entirely suitable for evaluating dementia‐prone cases, suggesting a need to develop appropriate criteria for evaluating prodromal DLB in iRBD subjects.^38^ Alternatively, the older age presentation with a marginally later onset of iRBD in dementia‐first converters might suggest different genetic and environmental triggers that predispose to parkinsonism‐first and dementia‐first conversion in RBD, respectively, albeit both PD and DLB were found to share the same Lewy fold synuclein aggregates in postmortem pathologies.^39^ A recent neuroimaging study also suggested a differential pattern of brain changes among the iRBD subjects who developed DLB compared to those who converted to PD.^40^ Overall, this study supported a differential trajectory course between parkinsonism‐first and dementia‐first neurodegeneration in iRBD.

Additionally, our study adopted a prospective matched cohort design to investigate the magnitude of neurodegenerative risk of iRBD compared to a non‐RBD group. We found a nearly 10‐time neurodegenerative risk in iRBD patients compared to non‐RBD subjects (adjusted HR = 9.6), which closely resembled the findings of a recent retrospective cohort study conducted in a real‐world setting (HR = 10.4).^41^ Our study is comparable to the studies from other Asian groups,^12^, ^13^, ^17^, ^18^, ^19^ suggesting that the conversion rate of RBD seemed to be somewhat lower compared to Caucasian patients in Europe and North America.^5^ The difference might be attributed to the methodological differences across studies, for example, different follow‐up intervals between visits. In our cohort, the 1.5–2‐year follow‐up interval might have led to a potential delay in α‐synucleinopathies diagnosis, especially during initial years. Additionally, the relatively low conversion rate observed in our study may be attributed to an insufficient follow‐up duration. Previous meta‐analyses have suggested that more phenoconversion events occur over longer periods, and our data also showed an increase in phenoconversion events over time.^21^ Nevertheless, there remained a possibility of potential cross‐ethnic and geographical differences that might underlie the disparate disease courses among different ethnic groups.^19^, ^42^, ^43^ These considerations might suggest the importance of accounting for ethnic and regional variability when designing neuroprotective trials for iRBD across diverse populations.

Our study had several clinical implications. First, our findings emphasize the importance of regular and individualized assessments of neurodegenerative features among iRBD patients. Individuals at high risk of phenoconversion—based on clinical profiles or biomarker findings—should undergo more frequent follow‐up, whereas those at lower risk may require less intensive monitoring. This risk‐based approach may help reduce patient burden and optimize clinical resources by minimizing unnecessary assessments, while still supporting early identification of neurodegenerative progression and potential referral for treatment, clinical trials, or preventive strategies. In this regard, future study and clinical practice should consider employing valid digital and other ambulatory devices to assist in clinical monitoring.^44^, ^45^ Second, our study comprehensively investigated the trajectories of motor and nonmotor clinical markers in iRBD patients compared to control subjects and suggested an accelerated nonlinear motor and global cognition deterioration course before the diagnosis of parkinsonism or dementia. The nonlinear trajectory course may help to determine the optimal time window for future neuroprotective intervention in these high‐risk subjects of α‐synucleinopathies.^46^ Given previous data suggesting an ~6% annual conversion rate in iRBD patients,^5^ very large sample sizes would be required for clinical trials. The nonlinear trajectory suggests that in real‐world prospective settings, researchers might recruit iRBD patients who have begun to show motor or cognitive symptoms for clinical trials, as they are at higher risk of rapid disease conversion over the subsequent few years. This approach might improve trial efficiency and reduce required sample sizes. Third, our study employed a prospective matched cohort design to investigate the magnitude of neurodegenerative risk of iRBD compared to a matched non‐RBD group. We observed an approximately 10‐time increased risk of phenoconversion to overt neurodegenerative diseases among iRBD patients relative to matched non‐RBD subjects. Our findings extend prior studies by offering a clear quantification of the magnitude of neurodegenerative vulnerability in iRBD. Fourth, the differential trajectories of neurodegeneration observed between parkinsonism‐first and dementia‐first phenoconversion suggested a need to facilitate the identification of individuals who may be at risk for differential neurodegenerative outcomes.

Some limitations in this study should be acknowledged. First, the interval between each regular assessment in our cohort was approximately 1.5–2 years, which might lead to some potential delays in the diagnosis of α‐synucleinopathies. However, the serial repeated measurement of a comprehensive series of motor and neurocognitive features have allowed us to delineate the trajectories of progression. Second, the limited sample size in some measurements, for example, UMSARS‐Part III, resulted in insufficient statistical power to detect association between some neurodegenerative markers and future phenoconversion. The overall modest sample size of the iRBD cohort has limited the estimation of the quantitative conversion risk for subtypes of α‐synucleinopathies. Third, there was differential proportion of missing data between groups, with non‐RBD subjects exhibiting higher missingness in some markers (Grooved Pegboard Test, Color Trail Test, and Reye‐Osterrieth Complex Figure). This was primarily because our study spanned the COVID‐19 pandemic, during which many non‐RBD control subjects were unable to complete the full set of evaluations due to limited face‐to‐face contact time. This differential missingness of the markers' data may introduce some potential bias of group comparisons in those markers between non‐RBD subjects and iRBD patients. Further studies with larger sample sizes are warranted to validate these findings and address potential biases from missing data. Fourth, the diagnosis of α‐synucleinopathies in this study was based primarily on clinical features, without the regular use of neuroimaging, such as brain magnetic resonance imaging (MRI) or dopamine transporter single‐photon emission computed tomography (DAT‐SPECT), which may limit diagnostic accuracy. Fifth, a potential limitation is selection bias arising from recruiting non‐RBD subjects from both the community and a sleep clinic, as community volunteers may differ in baseline health status compared to clinic patients. However, the two subgroups in our study were similar in future neurodegenerative risk and longitudinal marker changes. Finally, no α‐synucleinopathy events were observed in the non‐RBD control group during the follow‐up period. As a result, we were unable to estimate a HR specifically for α‐synucleinopathy outcomes. Future studies with longer follow‐up periods or larger sample sizes are warranted to address this limitation.

## Conclusion

This prospective matched cohort study found that the conversion rate of iRBD was 4.7% at 3 years, 17.9% at 5 years, 30.1% at 7 years, 55.6% at 10 years, and 79.5% at 13 years in the Hong Kong Chinese iRBD population. There was a nearly 10‐fold higher risk of future neurodegeneration in iRBD patients compared to non‐RBD subjects. Additionally, our study showed that the motor and cognitive functions exhibited nonlinear progressive trajectory patterns with a markedly accelerated deterioration course before the neurodegenerative diagnosis. These findings underscore the clinical importance of early identification and longitudinal monitoring of iRBD for timely intervention and improved risk stratification in the context of neurodegenerative disease progression.

## Author Roles

(1) Research project: A. Conception, B. Organization and acquisition of data, C. Confirm the diagnosis; (2) Statistical analysis: A. Design, B. Execution, C. Review and critique; (3) Manuscript: A. Writing of the first draft, B. Review and critique.

L.Z.: 1A, 1B, 2A, 2B, 2C, 3A, 3B

Y.P.L.: 1A, 1B, 2A, 2C, 3B

B.H.: 1B, 3B

J.W.: 1B, 3B

S.T.: 1B, 3B

Y.H.Y.: 1B, 3B

S.Y.G.: 1B, 3B

S.W.H.C.: 1C, 3B

J.W.Y.C.: 1C, 3B

M.W.M.Y.: 1B, 3B

J.C.C.T.: 1B, 3B

S.X.L.: 1B, 3B

S.P.L.: 1B, 3B

V.C.T.M.: 1C, 3B

K.K.Y.M.: 1C, 3B

A.Y.Y.C.: 1C, 3B

J.H.Z.: 1A, 1B, 2C, 3B

Y.K.W.: 1A, 1B, 1C, 2C, 3B

## Financial Disclosures of all Authors (for the Preceding 12 Months)

J.W. received grants from the National Natural Science Foundation of China (82401742) and Guangdong Basic and Applied Basic Research Foundation (2024A1515011349). J.W.Y.C. received grants from Takeda Inc. and travel support from Lundbeck HK limited for overseas conference. J.H.Z. received grants from the National Natural Science Foundation of China (82171476) and Health and Medical Research Fund (12131501). Y.K.W. received personal fees from Eisai for lecture and travel support from Lundbeck HK and Aculys Pharma, Inc, and participated in Takeda‐sponsored research study, which are outside the submitted work. All other authors reported no competing interests.

## Supporting information


**Table S1** Demographics, lifestyles, and Movement Disorder Society (MDS) likelihood ratio of prodromal Parkinson's disease (PD) (excluding RBD) at baseline among nonconverters, parkinsonism‐first and dementia‐first converters.
**Table S2** Neurodegenerative markers at baseline between parkinsonism‐first and dementia‐first converters.
**Table S3** The progression of neurodegenerative markers among nonconverters and parkinsonism‐first converters.
**Table S4** The progression of neurodegenerative markers among nonconverters and dementia‐first converters.
**Table S5** The correlations of neurodegenerative markers change with time among non‐RBD subjects and patients with isolated rapid eye movement sleep behavior disorder (iRBD).
**Table S6** Demographics, lifestyles, and clinical features at baseline between non‐RBD subjects from community and sleep clinic.
**Table S7** The progression of neurodegenerative markers among community and sleep clinic non‐RBD subjects.
**Table S8** Summary of neurodegenerative markers associated with parkinsonism‐ or dementia‐first phenoconversion.
**Figure S1** The data distributions of neurodegenerative markers. Abbreviations: ROCF, Rey‐Osterrieth Complex Figure Test; UMSARS‐Part III, Unified Multiple System Atrophy Rating Scale Part III; UPDRS‐Part III, Unified Parkinson's Disease Rating Scale Part III; HADS, Hospital Anxiety and Depression Scale; RBDQ‐HK, RBD questionnaire‐Hong Kong; ESS, the Epworth Sleepiness Scale; OIT, Olfactory Identification Test; HK‐MoCA, Hong Kong Montreal Cognitive Assessment.
**Figure S2** The dynamic changes in color trail and ROCF delay recall among groups during disease progression. Abbreviations: ROCF, Rey‐Osterrieth Complex Figure Test.
**Figure S3** The dynamic changes of OIT correct score total error of hue test among groups during disease progression. Abbreviations: OIT, Olfactory Identification Test.
**Figure S4** The dynamic changes in Grooved Pegboard Test (dominant and nondominant hands) among groups during disease progression.
**Figure S5** The dynamic changes in systolic and diastolic blood pressure drop among groups during disease progression.
**Figure S6** Kaplan–Meier curve for overall conversion rate in community and clinical control subjects.

## Data Availability

The data that support the findings of this study are available on request from the corresponding author. The data are not publicly available due to privacy or ethical restrictions.
