# Supplementary material for: Progression of Motor and Cognitive Functions in Isolated REM Sleep Behavior Disorder: A 7‐Year Prospective Matched Cohort Study
Source: Mov Disord. 2026 May 1;41(8):2086–95. doi: 10.1002/mds.70346 (PMC13518261; doi:10.1002/mds.70346)
Supplement: Supplementary file 1 [file MDS-41-2086-s001.docx]

**Supplementary Methods:**

**Subject recruitment criteria**

The inclusion criteria for patients with iRBD were: 1) confirmed with vPSG; 2) free of any neurodegenerative disorders at baseline; 3) RBD symptoms were not secondary to other sleep disorders, mental disorders, or medications. The inclusion criteria for non-RBD subjects were: 1) age at baseline was older than 50 years old; 2) absence of RBD features as confirmed by vPSG (DEBs and RSWA) at baseline; 3) free of any neurodegenerative disorders at baseline. Phasic and tonic mentalis electromyogram (EMG) activities, as well as phasic bilateral anterior tibialis EMG activities, were scored according to the American Academy of Sleep Medicine 2012 criteria. Detailed scoring procedures have been described in our previous publication.^1^ The threshold for RSWA was based on previous publications from the Sleep Innsbruck Barcelona (SINBAR) group.^1^^,^ ^2^ As for the non-RBD subjects, 47.5% them were recruited from the community, and 52.5% were recruited from our sleep clinic with predominant primary diagnosis of obstructive sleep apnea and/or insomnia.

This study was conducted in compliance with the Declaration of Helsinki and approved by the Joint Chinese University of Hong Kong-New Territories East Cluster Clinical Research Ethics Committee (CREC Ref. No: 2018.641). All subjects gave written informed consent.

**Measurements:**

Unified Parkinson’s Disease Rating Scale part III (UPDRS-part III) and the Grooved Pegboard test were used to assess motor function, finger and hand dexterity, and motor speed.^3-5^ Global cognitive function as well as executive and visuospatial function were assessed by Hong Kong Montreal cognitive assessment (HK-MoCA) and Color Trail Test, respectively.^6^^,^ ^7^ The Reye-Osterrieth Complex Figure were used to measure visuoconstructional ability and nonverbal memory.^8^ Olfactory function and color vision were assessed by the locally validated Olfactory Identification Test and Farmsworth-Munsell 100 hue test, respectively.^9^^,^ ^10^ UMSARS-part III was used to measure postural blood pressure change from supine to standing after 2 minutes. We included UMSARS-part III measure in the cohort considering that autonomic dysfunction is not unique to MSA but also occurs in patients with PD or DLB,^11^^,^ ^12^ and orthostatic hypotension is an important item in calculating the likelihood ratio for prodromal PD.^13^

The total likelihood ratio (LR) and probability of prodromal PD were calculated according to the updated Movement Disorder Society (MDS) research criteria (2019).^13^ The items measured in this study, except for RBD item, were used to calculate the total likelihood ratio and probability of prodromal PD. Other unmeasured items (e.g., gene mutation, substantia nigra hyperechogenicity, etc.) in this study were assigned a point of 1.0 in the calculation.

**Statistical analysis**

Continuous and categorical variables were summarized as mean ± standard deviation or n (%). Normality of continuous data was assessed using the Shapiro–Wilk test and Q–Q plots. Univariate analyses of continuous variables were performed using one-way ANOVA, independent t tests, or the Mann–Whitney U test, as appropriate. Categorical variables were analyzed using the chi-square or Fisher’s exact test. All continuous neurodegenerative variables were converted to z-scores for further analysis.

The Kaplan-Meier survival curve was employed to visually depict the cumulative incidence of neurodegeneration, and the log-rank test was used to compare differences in neurodegenerative risk between groups. The Cox proportional hazards regression models were applied to quantify the neurodegenerative risk between groups. The hazard ratio (HR) and 95% confidence interval (CI) were calculated. The proportional hazards assumption for the Cox model was evaluated using Schoenfeld residuals tests, confirming no violations (global p-values ranged from 0.14 to 0.31).

Longitudinal changes in neurodegenerative markers between groups were analyzed using generalized estimating equation (GEE) models as the assumptions of linear mixed-effects models (such as normality of residuals) were violated. GEE accounts for within-subject correlations in repeated measures without strict distributional assumptions. In our models, follow-up duration (with baseline coded as 0), group, and their interaction term (follow-up duration × group) were included, with age at baseline and sex as covariates. Clinical markers were the outcomes. P values of time*group interaction effect were further adjusted by Benjamini-Hochberg procedure to account for multiple comparisons.

To estimate the trajectory of neurodegenerative markers, generalized additive model was employed with the R Package “mgsv”.^14-16^ For each model, the clinical markers were set as dependent variable and backward time duration (the time of last follow-up or phenoconversion was as year 0, then back to the baseline (year -1, year -2, etc)) and individual random effect were entered as independent variables. The age at baseline and sex were covariates. Equation 1 presented a generalized form of the model, where s() denotes the application of a spline fit. The effective degree of freedom (EDF) was estimated by the models and represented the degree of non-linearity. EDF greater than 1 but less than 2 indicates a weakly non-linear relationship, while EDF greater than 2 indicates a highly non-linear relationship.

Neurodegenerative marker ~ s(backward time duration) + s(individual random effect) + age at baseline + sex	(1)

**Supplementary Table 1 Demographics, lifestyles, and MDS likelihood ratio of prodromal PD (excluding RBD) at** **baseline among nonconverters, parkinsonism-first and dementia-first converters.**

|  | **Nonconverters^1^**  **(n = 66)** | **Parkinsonism-first converters^2^ (n = 23)** | **Dementia-first converters^3^ (n = 16)** | ***p* value** | **Post hoc analysis^@^** |
| --- | --- | --- | --- | --- | --- |
| Age at baseline, mean (SD) | 65.0 (6.9) | 65.0 (6.0) | 70.2 (6.6) | **0.02^&^** | 1=2<3 |
| Age at onset, mean (SD) | 60.2 (7.5) | 59.5 (5.9) | 64.2 (6.9) | 0.084^&^ | NA |
| Age at phenoconversion, mean (SD) | - | 71.2 (6.0) | 77.4 (6.0) | **0.003^€^** | NA |
| Male, n (%) | 49 (74.2) | 27 (95.7) | 12 (75.0) | 0.077^€^ | NA |
| RBD duration at baseline since RBD onset, year, mean (SD) | 4.9 (2.9) | 5.5 (2.7) | 5.9 (3.1) | 0.32^€^ | NA |
| RBD duration at baseline since diagnosis, year, mean (SD) | 0.31 (1.3) | 0.39 (1.4) | 0.68 (1.5) | 0.76^€^ | NA |
| Follow-up duration, year, mean (SD) | 6.9 (3.2) | 6.2 (2.7) | 7.2 (3.1) | 0.59^&^ | NA |
| Phenoconversion duration since RBD onset, year, mean (SD) | - | 11.7 (4.0) | 13.2 (5.4) | 0.36^&^ | NA |
| Phenoconversion duration since RBD diagnosis, year, mean (SD) | - | 6.6 (3.4) | 7.9 (3.8) | 0.28^&^ | NA |
| Education, secondary or above, n (%) | 37 (56.1) | 17 (73.9) | 11 (68.8) | 0.26^&^ | NA |
| Lifestyle | n = 56 | n = 19 | n = 10 |  |  |
| Smoking |  |  |  |  |  |
| Never | 39 (69.6) | 15 (78.9) | 5 (50.0) | 0.31^€^ | NA |
| Former | 7 (12.5) | 1 (5.3) | 3 (30.0) | 0.19^€^ | NA |
| Current | 10 (17.9) | 3 (15.8) | 2 (20.0) | 1.0^€^ | NA |
| Alcohol drinking |  |  |  |  |  |
| Never | 28 (50.0) | 14 (73.7) | 5 (50.0) | 0.19^€^ | NA |
| Former | 6 (10.7) | 0 (0) | 2 (20.0) | 0.20^€^ | NA |
| Current | 22 (39.3) | 5 (26.3) | 3 (30.0) | 0.62^€^ | NA |
| Coffee consumption |  |  |  |  |  |
| Never | 23 (41.1) | 9 (47.4) | 6 (60.0) | 0.51^€^ | NA |
| Former | 0 (0) | 0 (0) | 0 (0) | NA | NA |
| Current | 33 (58.9) | 10 (52.6) | 4 (40.0) | 0.51^€^ | NA |
| Psychiatric disorders, lifetime | 21 (31.8) | 5 (21.7) | 2 (12.5) | 0.28^€^ | NA |
| Antidepressant usage, lifetime | 17 (25.8) | 5 (21.7) | 1 (6.3) | 0.291^€^ | NA |
| Constipation, lifetime | 27 (40.9) | 6 (37.5) | 6 (37.5) | 0.45^&^ | NA |
| **MDS likelihood ratio of prodromal PD (excluding RBD) at baseline** |  |  |  |  |  |
| Total estimated LR with log transformation | 0.76 (0.61) | 1.05 (0.6) | 0.62 (0.53) | **0.035^€^** | 1=3<2 |
| Total estimated probability | 0.13 (0.21) | 0.2 (0.23) | 0.12 (0.16) | 0.11^€^ | NA |
| **MDS likelihood ratio of prodromal PD (excluding RBD) at last follow-up or phenoconversion** |  |  |  |  |  |
| Total estimated LR with log transformation | 1.3 (0.78) | 1.9 (0.49) | 1.7 (0.82) | **<0.001^€^** | 1<3 |
| Total estimated probability | 0.35 (0.33) | 0.62 (0.25) | 0.59 (0.31) | **<0.001^€^** | 1<2=3 |

Data are presented as mean (standard deviation) or n (%). The bolded p values indicated < 0.05.

^&^ *p* value was estimated with one-way ANOVA or Chi-square test.

^€^ *p* value was estimated with nonparametric Mann-Whitney U test or Fisher's exact test.

^@^ Benjamini-Hochberg procedure was used to adjust p value (q-value), and q-value < 0.05 indicated statistically significant.

Abbreviations: SD, standard deviation; NA, not applicable.

**Supplementary Table 2 Neurodegenerative markers at baseline between** **parkinsonism-first and dementia-first converters.**

|  | **Parkinsonism-first converters (n = 23)** | **Dementia-first converters (n = 16)** | ***p* value** |
| --- | --- | --- | --- |
| HADS, total | 11.2 (6.4) | 10.3 (3.7) | 1.0 |
| ESS, total | 9.8 (4.4) | 6.5 (3.9) | **0.03** |
| RBDQ-HK | 46.9 (15.8) | 45.3 (25.3) | 0.50 |
| HK-MoCA | 25.4 (2.8) | 22.8 (2.1) | **0.004** |
| Color trail 2a, sec | 23.7 (17.3) | 18.8 (7.7) | 0.98 |
| Color trail 2b, sec | 121.9 (43.7) | 148.5 (64.9) | 0.35 |
| ROCF, copy | 32.7 (4.0) | 27.8 (12.6) | 0.29 |
| ROCF, immediate recall | 16.2 (8.3) | 8.0 (5.3) | **0.031** |
| ROCF, delay recall | 15.9 (7.3) | 9.8 (5.7) | 0.085 |
| Farnsworth Munsell 100 hue test, total error score | 185.9 (47.4) | 214.4 (70.3) | 0.53 |
| OIT, correct score | 1.2 (1.3) | 2.2 (1.6) | 0.07 |
| UPDRS-part III | 2.3 (1.9) | 2.6 (5.3) | 0.33 |
| Grooved Pegboard test, dominant, sec | 90.1 (22.3) | 85.0 (21.1) | 0.44 |
| Grooved Pegboard test, non-dominant sec | 91.8 (24.7) | 113.2 (90.3) | 0.44 |
| **UMSARS-part III** |  |  |  |
| Systolic Blood Pressure drop, mmHg | -0.22 (16.8) | -2.3 (15.0) | 0.86 |
| Diastolic Blood Pressure drop, mmHg | -5.0 (9.7) | -5.0 (6.0) | 0.73 |

Data are presented as mean (standard deviation). The bolded p values indicated < 0.05.

*p* value was estimated with nonparametric Mann-Whitney U test.

Abbreviations: SD, standard deviation; HADS, Hospital Anxiety and Depression Scale; ESS, the Epworth Sleepiness Scale; RBDQ-HK, RBD questionnaire-Hong Kong; HK-MoCA, Hong Kong Montreal cognitive assessment; ROCF, Rey-Osterrieth Complex Figure Test; OIT, Olfactory Identification test; UPDRS-part III, Unified Parkinson's Disease Rating Scale part III; UMSARS-part III, Unified Multiple System Atrophy Rating Scale part III.

**Supplementary Table 3 The progression of neurodegenerative markers among nonconverters and parkinsonism-first converters.**

|  | **Baseline** | |  | **Time effect** | |  | **Group effect** | |  | **Time * Group interaction** | | |
| --- | --- | --- | --- | --- | --- | --- | --- | --- | --- | --- | --- | --- |
|  | **Nonconverters (n = 66)** | **Parkinsonism-first converters (n = 23)** |  | **Annual change** | ***p* value** |  | **Annual change** | ***p* value** |  | **Annual change** | ***p* value** | ***p_adjusted_* value** |
| HADS, total, z-score, mean (SD) | 0.09 (1) | 0.23 (1.07) |  | 0.01 (-0.03, 0.04) | 0.72 |  | 0.17 (-0.33, 0.67) | 0.50 |  | -0.02 (-0.10, 0.05) | 0.54 | 0.75 |
| ESS, total, z-score, mean (SD) | 0.03 (0.96) | 0.14 (0.88) |  | -0.03 (-0.06, 0.01) | 0.13 |  | 0.18 (-0.23, 0.58) | 0.39 |  | -0.01 (-0.09, 0.06) | 0.72 | 0.82 |
| RBDQ-HK, total, z-score, mean (SD) | 0.74 (0.74) | 0.91 (0.75) |  | **-0.05 (-0.08, -0.02)** | **<0.001** |  | 0.09 (-0.22, 0.40) | 0.57 |  | -0.05 (-0.12, 0.01) | 0.09 | 0.48 |
| HK-MoCA, total, z- score, mean (SD) | 0.05 (0.66) | 0.12 (0.9) |  | **-0.03 (-0.06, 0.00)** | **0.03** |  | -0.02 (-0.37, 0.33) | 0.91 |  | 0.02 (-0.04, 0.07) | 0.61 | 0.75 |
| Color trail 2a, sec, z-score, mean (SD) | -0.16 (0.53) | 0.05 (1.01) |  | **0.03 (0.01, 0.06)** | **0.02** |  | 0.11 (-0.25, 0.47) | 0.55 |  | -0.01 (-0.07, 0.05) | 0.79 | 0.84 |
| Color trail 2b, sec, z-score, mean (SD) | -0.26 (0.77) | -0.19 (0.78) |  | 0.06 (0.02, 0.09) | **<0.001** |  | 0.17 (-0.16, 0.51) | 0.32 |  | -0.04 (-0.09, 0.01) | 0.15 | 0.48 |
| ROCF, copy, z-score, mean (SD) | 0.06 (0.77) | 0.11 (0.77) |  | 0.00 (-0.04, 0.03) | 0.95 |  | -0.02 (-0.40, 0.37) | 0.94 |  | -0.01 (-0.10, 0.08) | 0.89 | 0.89 |
| ROCF, immediate recall, z-score, mean (SD) | -0.15 (0.91) | 0.02 (1.04) |  | -0.01 (-0.05, 0.03) | 0.63 |  | -0.10 (-0.62, 0.42) | 0.70 |  | **0.08 (0.02, 0.13)** | **0.01** | 0.08 |
| ROCF, delay recall, z-score, mean (SD) | -0.1 (0.9) | 0.02 (0.96) |  | 0.01 (-0.03, 0.06) | 0.52 |  | -0.03 (-0.53, 0.47) | 0.91 |  | 0.04 (-0.03, 0.11) | 0.30 | 0.60 |
| Farnsworth Munsell 100 hue test, total error score, z-score, mean (SD) | -0.08 (1) | -0.15 (0.53) |  | 0.00 (-0.04, 0.04) | 0.93 |  | -0.20 (-0.59, 0.20) | 0.32 |  | 0.04 (-0.02, 0.10) | 0.15 | 0.48 |
| OIT, correct score, z-score, mean (SD) | -0.12 (0.78) | -0.59 (0.7) |  | **-0.04 (-0.07, -0.01)** | **<0.001** |  | **-0.45 (-0.75, -0.16)** | **<0.001** |  | 0.02 (-0.03, 0.07) | 0.45 | 0.72 |
| UPDRS-part III, z-score, mean (SD) | -0.29 (0.59) | -0.41 (0.32) |  | **0.07 (0.04, 0.11)** | **<0.001** |  | 0.34 (-0.07, 0.76) | 0.10 |  | **0.14 (0.03, 0.25)** | **0.01** | 0.08 |
| Grooved Pegboard test, dominant, sec, z-score, mean (SD) | -0.29 (0.94) | 0.15 (0.76) |  | **0.05 (0.02, 0.08)** | **<0.001** |  | 0.62 (0.25, 0.99) | **<0.001** |  | 0.02 (-0.02, 0.07) | 0.30 | 0.60 |
| Grooved Pegboard test, non-dominant sec, z-score, mean (SD) | -0.32 (0.71) | -0.02 (0.69) |  | **0.05 (0.03, 0.08)** | **<0.001** |  | **0.47 (0.06, 0.88)** | **0.02** |  | 0.03 (-0.04, 0.09) | 0.42 | 0.72 |
| **UMSARS-part III** |  |  |  |  |  |  |  |  |  |  |  |  |
| Systolic Blood Pressure drop, mmHg, z-score, mean (SD) | -0.15 (0.77) | -0.13 (1.01) |  | - | - |  | -0.05 (-0.37, 0.26) | 0.74 |  | - | - |  |
| Diastolic Blood Pressure drop, mmHg, z-score, mean (SD) | -0.19 (0.9) | -0.38 (1.12) |  | - | - |  | -0.14 (-0.44, 0.15) | 0.34 |  | - | - |  |
| **MDS likelihood ratio of prodromal PD (excluding RBD)** | | |  |  |  |  |  |  |  |  |  |  |
| Total estimated LR with log transformation | 0.76 (0.61) | 1.05 (0.6) |  | **0.05 (0.02, 0.07)** | **<0.001** |  | **0.30 (0.02, 0.58)** | **0.03** |  | 0.01 (-0.03, 0.06) | 0.56 | 0.75 |
| Total estimated probability | 0.13 (0.21) | 0.2 (0.23) |  | **0.02 (0.02, 0.03)** | **<0.001** |  | 0.08 (-0.03, 0.18) | 0.14 |  | 0.01 (-0.01, 0.03) | 0.27 | 0.60 |

The models were generated by generalized estimating equation model adjusting for age at baseline and gender. Nonconverters were as the reference in the models. Benjamini-Hochberg procedure was used to adjust p value.

Abbreviations: SD, standard deviation; HADS, Hospital Anxiety and Depression Scale; ESS, the Epworth Sleepiness Scale; RBDQ-HK, RBD questionnaire-Hong Kong; HK-MoCA, Hong Kong Montreal cognitive assessment; ROCF, Rey-Osterrieth Complex Figure Test; OIT, Olfactory Identification test; UPDRS-part III, Unified Parkinson's Disease Rating Scale part III; UMSARS-part III, Unified Multiple System Atrophy Rating Scale part III.

**Supplementary Table 4 The progression of neurodegenerative markers among nonconverters and dementia-first converters.**

|  | **Baseline** | |  | **Time effect** | |  | **Group effect** | |  | **Time * Group interaction** | | |
| --- | --- | --- | --- | --- | --- | --- | --- | --- | --- | --- | --- | --- |
|  | **Nonconverters (n = 66)** | **Dementia-first converters (n = 16)** |  | **Annual change** | ***p* value** |  | **Annual change** | ***p* value** |  | **Annual change** | ***p* value** | ***p_adjusted_* value** |
| HADS, total, z-score, mean (SD) | 0.09 (1) | 0.08 (0.62) |  | 0.00 (-0.03, 0.04) | 0.8 |  | -0.20 (-0.72, 0.32) | 0.46 |  | 0.04 (-0.07, 0.16) | 0.43 | 0.43 |
| ESS, total, z-score, mean (SD) | 0.03 (0.96) | -0.51 (0.78) |  | -0.03 (-0.07, 0.01) | 0.14 |  | **-0.56 (-1.00, -0.13)** | **0.01** |  | 0.05 (-0.05, 0.16) | 0.32 | 0.39 |
| RBDQ-HK, total, z-score, mean (SD) | 0.74 (0.74) | 0.84 (1.21) |  | **-0.05 (-0.08, -0.02)** | **<0.001** |  | 0.07 (-0.44, 0.57) | 0.79 |  | -0.04 (-0.13, 0.05) | 0.42 | 0.43 |
| HK-MoCA, total, z- score, mean (SD) | 0.05 (0.66) | -0.75 (0.69) |  | -0.03 (-0.06, 0.00) | 0.04 |  | -0.39 (-0.88, 0.10) | 0.12 |  | **-0.14 (-0.22, -0.06)** | **<0.001** | **<0.001** |
| Color trail 2a, sec, z-score, mean (SD) | -0.16 (0.53) | -0.24 (0.45) |  | 0.03 (0.00, 0.06) | 0.03 |  | 0.08 (-0.61, 0.76) | 0.83 |  | **0.18 (0.03, 0.33)** | **0.02** | **0.04** |
| Color trail 2b, sec, z-score, mean (SD) | -0.26 (0.77) | 0.29 (1.15) |  | 0.05 (0.02, 0.09) | **<0.001** |  | 0.25 (-0.74, 1.25) | 0.62 |  | **0.16 (0.02, 0.30)** | **0.02** | **0.04** |
| ROCF, copy, z-score, mean (SD) | 0.06 (0.77) | -0.84 (2.41) |  | 0.00 (-0.04, 0.03) | 0.94 |  | -0.30 (-1.16, 0.56) | 0.49 |  | -0.13 (-0.32, 0.06) | 0.18 | 0.24 |
| ROCF, immediate recall, z-score, mean (SD) | -0.15 (0.91) | -1.01 (0.67) |  | -0.01 (-0.05, 0.03) | 0.64 |  | -0.59 (-1.20, 0.01) | 0.05 |  | -0.04 (-0.12, 0.04) | 0.35 | 0.40 |
| ROCF, delay recall, z-score, mean (SD) | -0.1 (0.9) | -0.78 (0.75) |  | 0.01 (-0.03, 0.06) | 0.50 |  | -0.45 (-1.09, 0.19) | 0.17 |  | -0.07 (-0.15, 0.01) | 0.07 | 0.12 |
| Farnsworth Munsell 100 hue test, total error score, z-score, mean (SD) | -0.08 (1) | 0.17 (0.79) |  | 0.00 (-0.04, 0.04) | 0.94 |  | 0.08 (-0.54, 0.69) | 0.81 |  | **0.15 (0.05, 0.26)** | **<0.001** | **<0.001** |
| OIT, correct score, z-score, mean (SD) | -0.12 (0.78) | -0.05 (0.89) |  | **-0.04 (-0.07, -0.01)** | **0.01** |  | 0.18 (-0.19, 0.55) | 0.34 |  | **-0.08 (-0.13, -0.03)** | **<0.001** | **<0.001** |
| UPDRS-part III, z-score, mean (SD) | -0.29 (0.59) | -0.36 (0.89) |  | **0.07 (0.04, 0.11)** | **<0.001** |  | -0.28 (-0.83, 0.27) | 0.32 |  | **0.15 (0.04, 0.25)** | **0.01** | **0.03** |
| Grooved Pegboard test, dominant, sec, z-score, mean (SD) | -0.29 (0.94) | -0.03 (0.72) |  | **0.05 (0.02, 0.08)** | **<0.001** |  | 0.22 (-0.57, 1.01) | 0.58 |  | 0.10 (-0.02, 0.22) | 0.11 | 0.16 |
| Grooved Pegboard test, non-dominant sec, z-score, mean (SD) | -0.32 (0.71) | 0.58 (2.52) |  | **0.05 (0.02, 0.07)** | **<0.001** |  | 0.00 (-1.04, 1.05) | 0.99 |  | 0.15 (-0.03, 0.34) | 0.11 | 0.16 |
| **UMSARS-part III** |  |  |  |  |  |  |  |  |  |  |  |  |
| Systolic Blood Pressure drop, mmHg, z-score, mean (SD) | -0.15 (0.77) | -0.26 (0.9) |  | - | - |  | **0.54 (0.13, 0.95)** | **0.01** |  | - | - |  |
| Diastolic Blood Pressure drop, mmHg, z-score, mean (SD) | -0.19 (0.9) | -0.38 (0.69) |  | - | - |  | **0.60 (0.24, 0.96)** | **<0.001** |  | - | - |  |
| **MDS likelihood ratio of prodromal PD (excluding RBD)** | | |  |  |  |  |  |  |  |  |  |  |
| Total estimated LR with log transformation | 0.76 (0.61) | 0.62 (0.53) |  | **0.04 (0.02, 0.07)** | **<0.001** |  | **-0.40 (-0.68, -0.12)** | **<0.001** |  | **0.13 (0.08, 0.18)** | **<0.001** | **<0.001** |
| Total estimated probability | 0.13 (0.21) | 0.12 (0.16) |  | **0.02 (0.01, 0.03)** | **<0.001** |  | **-0.17 (-0.27, -0.07)** | **<0.001** |  | **0.05 (0.03, 0.07)** | **<0.001** | **<0.001** |

The models were generated by generalized estimating equation model adjusting for age at baseline and gender. Nonconverters were as the reference in the models. Benjamini-Hochberg procedure was used to adjust p value.

Abbreviations: SD, standard deviation; HADS, Hospital Anxiety and Depression Scale; ESS, the Epworth Sleepiness Scale; RBDQ-HK, RBD questionnaire-Hong Kong; HK-MoCA, Hong Kong Montreal cognitive assessment; ROCF, Rey-Osterrieth Complex Figure Test; OIT, Olfactory Identification test; UPDRS-part III, Unified Parkinson's Disease Rating Scale part III; UMSARS-part III, Unified Multiple System Atrophy Rating Scale part III.

**Supplementary Table 5 The correlations of neurodegenerative markers changes with time among non-RBD subjects and patients with iRBD.**

|  | **Non-RBD subjects** | |  | **iRBD patients** | |
| --- | --- | --- | --- | --- | --- |
|  | **EDF** | ***p* value** |  | **EDF** | ***p* value** |
| HK-MoCA, total, z- score | **4.5** | **0.02** |  | **2.1** | **<0.001** |
| UPDRS-part III, z-score | **1.0** | **0.008** |  | **2.7** | **<0.001** |
| Color trail 2a, sec, z-score | 1.8 | 0.40 |  | **1.3** | **0.03** |
| Color trail 2b, sec, z-score | 1.0 | 0.96 |  | **1.0** | **0.002** |
| ROCF, delay recall, z-score | 1.0 | 0.90 |  | 1.0 | 0.44 |
| OIT, correct score, z-score | 1.0 | 0.70 |  | **1.6** | **0.02** |
| FarnswortheMunsell 100 hue test, total error score, z-score | 1.0 | 0.36 |  | **1.0** | **0.004** |
| Grooved Pegboard test, dominant, sec | 1.0 | 0.79 |  | **1.3** | **<0.001** |
| Grooved Pegboard test, non-dominant, sec | 1.0 | 1.0 |  | **1.5** | **<0.001** |
| **UMSARS-part III** |  |  |  |  |  |
| Systolic Blood Pressure drop, mmHg, z-score | 1.0 | 0.14 |  | **1.0** | **0.003** |
| Diastolic Blood Pressure drop, mmHg, z-score | 1.2 | 0.43 |  | **1.6** | **0.001** |

The models were generated by Generalized additive models adjusting for age at baseline and gender. Individual subjects were as random effect in the models. The bolded p values indicated < 0.05.

Abbreviations: EDF = effective degrees of freedom; iRBD, isolated REM sleep behavior disorder; HK-MoCA, Hong Kong Montreal cognitive assessment; ROCF, Rey-Osterrieth Complex Figure Test; OIT, Olfactory Identification test; UPDRS-part III, Unified Parkinson's Disease Rating Scale part III; UMSARS-part III, Unified Multiple System Atrophy Rating Scale part III.

**Supplementary Table 6** **Demographics, lifestyles, and clinical features at baseline between non-RBD subjects from community and sleep clinic.**

|  | Community non-RBD subjects (n = 48) | Clinical non-RBD subjects (n = 53) | *p* value |
| --- | --- | --- | --- |
| Age at baseline | 68.2 ± 10.5 | 65.3 ± 8.2 | 0.13^&^ |
| Male | 36 (75.0) | 35 (66.0) | 0.33^&^ |
| Follow-up duration, year | 6.4 ± 2.7 | 7.8 ± 2.9 | **0.012^&^** |
| Education, secondary or above | 27 (56.3) | 36 (67.9) | 0.23^&^ |
| Sleep disorders |  |  |  |
| OSA | - | 50 (94.3) | - |
| OSA patients with treatment | - | 39 (78.0) | - |
| Insomnia | - | 5 (9.4) | - |
| Lifestyle |  |  |  |
| Smoking | n = 45 | n = 42 |  |
| Never | 36 (80.0) | 37 (88.1) | 0.31^&^ |
| Former | 6 (13.3) | 4 (9.5) | 0.74^€^ |
| Current | 3 (6.7) | 1 (2.4) | 0.62^€^ |
| Alcohol drinking | n = 44 | n = 42 |  |
| Never | 25 (56.8) | 21 (50.0) | 0.53^&^ |
| Former | 1 (2.3) | 3 (7.1) | 0.36^€^ |
| Current | 18 (40.9) | 18 (42.9) | 0.86^&^ |
| Coffee assumption | n = 45 | n = 42 |  |
| Never | 24 (53.3) | 15 (35.7) | 0.10^&^ |
| Former | 0 (0) | 1 (2.4) | 0.48^€^ |
| Current | 21 (21) | 26 (61.9) | 0.15^&^ |
| Psychiatric disorders, lifetime | 4 (8.3) | 17 (32.1) | **0.003^&^** |
| Antidepressant usage, lifetime | 3 (6.3) | 15 (28.3) | **0.004^&^** |
| HADS, total score | 7.7 (5.9)  n = 47 | 9.7 (6.3)  n = 49 | 0.11^€^ |
| Constipation, lifetime | 8 (16.7) | 10 (18.9) | 0.77^&^ |

Data are presented as mean (standard deviation) or n (%). The bolded p values indicated < 0.05.

^&^ *p* value was estimated with independent t-test or Chi-square test.

^€^ *p* value was estimated with nonparametric Mann-Whitney U test or Fisher's exact test. Abbreviations: RBD, REM sleep behavior disorder; HADS, Hospital Anxiety and Depression Scale; OSA, obstructive sleep apnea.

**Supplementary Table 7 The progression of neurodegenerative markers among community and sleep clinic non-RBD subjects.**

|  | **Baseline** | |  | **Time effect** | |  | **Group effect** | |  | **Time * Group interaction** | |  |
| --- | --- | --- | --- | --- | --- | --- | --- | --- | --- | --- | --- | --- |
|  | **Community non-RBD subjects (n = 37)** | **Clinical non-RBD subjects (n = 43)** |  | **Annual change** | ***p* value** |  | **Annual change** | ***p* value** |  | **Annual change** | ***p* value** | |
| HADS, total, z-score, mean (SD) | -0.39 ± 0.97 | 0.05 ± 1.06 |  | 0.04 (-0.04, 0.12) | 0.38 |  | 0.41 (-0.02, 0.84) | 0.06 |  | -0.04 (-0.14, 0.06) | 0.42 | |
| ESS, total, z-score, mean (SD) | 0.07 ± 0.95 | 0.33 ± 1.12 |  | 0 (-0.07, 0.07) | 0.98 |  | 0.15 (-0.31, 0.61) | 0.53 |  | 0 (-0.09, 0.08) | 0.95 | |
| RBDQ-HK, total, z-score, mean (SD) | -1.04 ± 0.3 | -0.87 ± 0.39 |  | -0.01 (-0.03, 0.01) | 0.25 |  | 0.14 (0, 0.28) | 0.05 |  | 0 (-0.03, 0.02) | 0.81 | |
| HK-MoCA, total, z- score, mean (SD) | 0.24 ± 0.78 | 0.02 ± 0.92 |  | -0.04 (-0.09, 0) | 0.07 |  | **-0.31 (-0.58, -0.04)** | **0.02** |  | 0.05 (-0.01, 0.11) | 0.08 | |
| Color trail 2a, sec, z-score, mean (SD) | -0.3 ± 0.47 | -0.23 ± 0.59 |  | 0.01 (-0.04, 0.07) | 0.60 |  | 0.07 (-0.15, 0.3) | 0.51 |  | 0.03 (-0.07, 0.13) | 0.57 | |
| Color trail 2b, sec, z-score, mean (SD) | -0.23 ± 0.82 | -0.21 ± 0.83 |  | 0.07 (-0.1, 0.24) | 0.43 |  | 0.14 (-0.17, 0.46) | 0.37 |  | -0.09 (-0.28, 0.1) | 0.34 | |
| ROCF, copy, z-score, mean (SD) | 0.26 ± 0.52 | 0.15 ± 0.65 |  | -0.31 (-0.69, 0.08) | 0.12 |  | -0.11 (-0.39, 0.16) | 0.42 |  | 0.27 (-0.12, 0.67) | 0.17 | |
| ROCF, immediate recall, z-score, mean (SD) | -0.03 ± 0.9 | 0.05 ± 0.86 |  | -0.03 (-0.22, 0.15) | 0.72 |  | 0.2 (-0.15, 0.55) | 0.27 |  | 0.01 (-0.18, 0.21) | 0.88 | |
| ROCF, delay recall, z-score, mean (SD) | -0.12 ± 0.83 | -0.02 ± 0.85 |  | -0.05 (-0.19, 0.09) | 0.46 |  | 0.19 (-0.14, 0.53) | 0.26 |  | 0.06 (-0.1, 0.21) | 0.48 | |
| Farnsworth Munsell 100 hue test, total error score, z-score, mean (SD) | 0.08 ± 0.9 | -0.13 ± 0.81 |  | -0.02 (-0.1, 0.06) | 0.57 |  | 0.02 (-0.32, 0.36) | 0.92 |  | 0.02 (-0.06, 0.11) | 0.59 | |
| OIT, correct score, z-score, mean (SD) | 0.68 ± 0.89 | 0.68 ± 0.91 |  | 0 (-0.06, 0.06) | 0.96 |  | -0.27 (-0.59, 0.05) | 0.10 |  | 0 (-0.07, 0.07) | 0.97 | |
| UPDRS-part III, z-score, mean (SD) | -0.6 ± 0.4 | -0.51 ± 0.44 |  | **0.06 (0.03, 0.09)** | **<0.001** |  | 0.1 (-0.11, 0.31) | 0.37 |  | -0.02 (-0.06, 0.03) | 0.45 | |
| Grooved Pegboard test, dominant, sec, z-score, mean (SD) | -0.25 ± 0.93 | -0.34 ± 0.56 |  | 0.03 (-0.1, 0.16) | 0.61 |  | 0.12 (-0.18, 0.41) | 0.44 |  | -0.02 (-0.15, 0.12) | 0.82 | |
| Grooved Pegboard test, non-dominant sec, z-score, mean (SD) | -0.25 ± 0.5 | -0.26 ± 0.59 |  | 0.04 (-0.05, 0.13) | 0.41 |  | 0.15 (-0.05, 0.35) | 0.13 |  | -0.04 (-0.14, 0.05) | 0.39 | |
| **UMSARS-part III** |  |  |  |  |  |  |  |  |  |  |  | |
| Systolic Blood Pressure drop, mmHg, z-score, mean (SD) | -0.56 ± 0.7 | -0.33 ± 0.66 |  | 0.05 (-0.02, 0.12) | 0.17 |  | -0.15 (-0.56, 0.25) | 0.45 |  | -0.02 (-0.11, 0.06) | 0.62 | |
| Diastolic Blood Pressure drop, mmHg, z-score, mean (SD) | -0.58 ± 0.78 | -0.32 ± 0.91 |  | 0.06 (-0.01, 0.13) | 0.09 |  | 0 (-0.52, 0.51) | 0.99 |  | -0.02 (-0.12, 0.07) | 0.65 | |
| **MDS likelihood ratio of prodromal PD (excluding RBD)** | | |  |  |  |  |  |  |  |  |  | |
| Total estimated LR with log transformation | 0.39 ± 0.53 | 0.48 ± 0.49 |  | **0.04 (0.01, 0.07)** | **0.02** |  | 0.18 (-0.01, 0.36) | 0.06 |  | -0.02 (-0.06, 0.02) | 0.33 | |
| Total estimated probability | 0.07 ± 0.19 | 0.07 ± 0.12 |  | **0.01 (0, 0.03)** | **0.02** |  | 0.03 (-0.03, 0.09) | 0.30 |  | 0 (-0.02, 0.01) | 0.56 | |

The models were generated by generalized estimating equation model adjusting for age at baseline and gender. Community non-RBD subjects were as the reference in the models.

Abbreviations: SD, standard deviation; HADS, Hospital Anxiety and Depression Scale; ESS, the Epworth Sleepiness Scale; RBDQ-HK, RBD questionnaire-Hong Kong; HK-MoCA, Hong Kong Montreal cognitive assessment; ROCF, Rey-Osterrieth Complex Figure Test; OIT, Olfactory Identification test; UPDRS-part III, Unified Parkinson's Disease Rating Scale part III; UMSARS-part III, Unified Multiple System Atrophy Rating Scale part III.

**Supplementary table 8 Summary of neurodegenerative markers associated with** **parkinsonism- or dementia-first phenoconversion.**

| Parkinsonism-first phenocovnersion | Dementia-first phenoconversion |
| --- | --- |
| - Motor function (UPDRS-part III) | - Cognition (HK-MoCA) - Color vision function (Farmsworth-Munsell 100 hue test) - Motor function (UPDRS-part III) - Olfactory function (Olfactory Identification Test) |

Abbreviations: HK-MoCA, Hong Kong Montreal cognitive assessment; UPDRS-part III, Unified Parkinson's Disease Rating Scale part III.

Supplementary Fig. 1 The data distributions of neurodegenerative markers. Abbreviations: ROCF, Rey-Osterrieth Complex Figure Test; UMSARS-part III, Unified Multiple System Atrophy Rating Scale part III; UPDRS-part III, Unified Parkinson's Disease Rating Scale part III; HADS, Hospital Anxiety and Depression Scale; RBDQ-HK, RBD questionnaire-Hong Kong; ESS, the Epworth Sleepiness Scale; OIT, Olfactory Identification test; HK-MoCA, Hong Kong Montreal cognitive assessment.

Supplementary Fig. 2 The dynamic changes of color trail and ROCF delay recall among groups during disease progression. Abbreviations: ROCF, Rey-Osterrieth Complex Figure Test.

Supplementary Fig. 3 The dynamic changes of OIT correct score total error of hue test among groups during disease progression. Abbreviations: OIT, Olfactory Identification test.

Supplementary Fig. 4 The dynamic changes of Grooved pegboard test (dominant and non-dominant hands) among groups during disease progression.

Supplementary Fig. 5 The dynamic changes of systolic and diastolic blood pressure drop among groups during disease progression.

Supplementary Fig. 6 Kaplan-Meier curve for overall conversion rate in community and clinical control subjects.

**References:**

1.	Liu Y, Zhang J, Chau SWH, et al. Evolution of Prodromal REM Sleep Behavior Disorder to Neurodegeneration: A Retrospective Longitudinal Case-Control Study. Neurology 2022;99:e627–e637.

2.	Frauscher B, Iranzo A, Gaig C, et al. Normative EMG values during REM sleep for the diagnosis of REM sleep behavior disorder. Sleep 2012;35:835–847.

3.	The Unified Parkinson's Disease Rating Scale (UPDRS): status and recommendations. Mov Disord 2003;18:738–750.

4.	Bryden PJ, Roy EA. A new method of administering the Grooved Pegboard Test: Performance as a function of handedness and sex. Brain and Cognition 2005;58:258–268.

5.	Wang J, Huang B, Zhou L, et al. Visuospatial dysfunction predicts dementia-first phenoconversion in isolated REM sleep behaviour disorder. J Neurol Neurosurg Psychiatry 2024:jnnp–2024–333865.

6.	Wong A, Xiong YY, Kwan PW, et al. The validity, reliability and clinical utility of the Hong Kong Montreal Cognitive Assessment (HK-MoCA) in patients with cerebral small vessel disease. Dement Geriatr Cogn Disord 2009;28:81–87.

7.	Lee TM, Chan CC. Are trail making and color trails tests of equivalent constructs? J Clin Exp Neuropsychol 2000;22:529–534.

8.	Shin MS, Park SY, Park SR, Seol SH, Kwon JS. Clinical and empirical applications of the Rey-Osterrieth Complex Figure Test. Nat Protoc 2006;1:892–899.

9.	Chan A, Tam J, Murphy C, Chiu H, Lam L. Utility of olfactory identification test for diagnosing Chinese patients with Alzheimer's disease. J Clin Exp Neuropsychol 2002;24:251–259.

10.	Farnsworth D. The Farnsworth-Munsell 100-Hue and Dichotomous Tests for Color Vision*. J Opt Soc Am 1943;33:568–578.

11.	Chen Z, Li G, Liu J. Autonomic dysfunction in Parkinson’s disease: Implications for pathophysiology, diagnosis, and treatment. Neurobiology of Disease 2020;134:104700.

12.	Thaisetthawatkul P, Boeve BF, Benarroch EE, et al. Autonomic dysfunction in dementia with Lewy bodies. Neurology 2004;62:1804–1809.

13.	Heinzel S, Berg D, Gasser T, Chen H, Yao C, Postuma RB. Update of the MDS research criteria for prodromal Parkinson's disease. Mov Disord 2019;34:1464–1470.

14.	Lucey BP, Wisch J, Boerwinkle AH, et al. Sleep and longitudinal cognitive performance in preclinical and early symptomatic Alzheimer's disease. Brain 2021;144:2852–2862.

15.	Nedelska Z, Schwarz CG, Lesnick TG, et al. Association of Longitudinal β-Amyloid Accumulation Determined by Positron Emission Tomography With Clinical and Cognitive Decline in Adults With Probable Lewy Body Dementia. JAMA Netw Open 2019;2:e1916439.

16.	Wood SN. Generalized Additive Models: An Introduction with R (2nd edition). Chapman and Hall/CRC 2017.
